# Kinematic decomposition and classification of octopus arm movements

**DOI:** 10.3389/fncom.2013.00060

**Published:** 2013-05-24

**Authors:** Ido Zelman, Myriam Titon, Yoram Yekutieli, Shlomi Hanassy, Binyamin Hochner, Tamar Flash

**Affiliations:** ^1^Department of Computer Science and Applied Mathematics, Weizmann Institute of ScienceRehovot, Israel; ^2^General Motors, Advanced Technical Center - IsraelHerzliya, Israel; ^3^Department of Computer Science, Hadassah Academic CollegeJerusalem, Israel; ^4^Department of Neurobiology and Interdisciplinary Center for Neural Computation, Hebrew UniversityJerusalem, Israel

**Keywords:** octopus, motion analysis, kinematic motion primitives (kMPs), 3D reconstruction, muscular hydrostat

## Abstract

The octopus arm is a muscular hydrostat and due to its deformable and highly flexible structure it is capable of a rich repertoire of motor behaviors. Its motor control system uses planning principles and control strategies unique to muscular hydrostats. We previously reconstructed a data set of octopus arm movements from records of natural movements using a sequence of 3D curves describing the virtual backbone of arm configurations. Here we describe a novel representation of octopus arm movements in which a movement is characterized by a pair of surfaces that represent the curvature and torsion values of points along the arm as a function of time. This representation allowed us to explore whether the movements are built up of elementary kinematic units by decomposing each surface into a weighted combination of 2D Gaussian functions. The resulting Gaussian functions can be considered as motion primitives at the kinematic level of octopus arm movements. These can be used to examine underlying principles of movement generation. Here we used combination of such kinematic primitives to decompose different octopus arm movements and characterize several movement prototypes according to their composition. The representation and methodology can be applied to the movement of any organ which can be modeled by means of a continuous 3D curve.

## Introduction

Octopuses are considered to be among the most developed and intelligent animals in the invertebrate kingdom, where at least part of their skills can be attributed to the high maneuverability of their arms and the capacity of the peripheral nervous system to process sensory information and control arm movements. The octopus uses its arms for various tasks such as locomotion, food gathering, hunting, and sophisticated object manipulation (Wells and Wells, 1957; Fiorito et al., 1990; Mather, 1998). The versatile and adaptive nature of octopus movements is mainly due to the flexible nature of the octopus arms which do not contain any rigid skeleton. The octopus arm is a muscular hydrostat built of closely packed arrays of muscle fibers organized in three main muscle groups: parallel, perpendicular, and helical that runs obliquely to the long axis (Matzner et al., 2000). A constant volume constraint that holds for muscular hydrostats allows forces to be transferred between the longitudinal and the transverse muscle groups. The movements of a muscular hydrostat are based on combinations of four elementary movements that can occur at any location: elongation, shortening, torsion, and bending (Kier and Smith, 1985). Therefore, both structural support and force transmission are achieved through the arm's musculature, such that the biomechanical principles governing octopus arm movements differ from those operating in arms with a rigid skeletal support.

The octopus nervous system is divided into a central and peripheral nervous systems. Axial nerve cords are projecting from the brain along the center of each arm, and the peripheral neurons located in the axial nerve cords are organized into an extensive nervous system comprising both sensory and motor circuits (Young, 1971). Behavioral studies suggest that the nerve cord circuitry and the peripheral components play a major role in the control of the complex actions performed by octopus arms (Altman, 1971; Wells, 1978).

Analyses of octopus reaching (Gutfreund et al., 1996, 1998; Sumbre et al., 2001; Yekutieli et al., 2005a,b) and fetching movements (Sumbre et al., 2001, 2005, 2006) have revealed some control principles that underlie movement generation. During reaching a bend point propagates along the arm following an invariant velocity profile. Fetching movements use a vertebrate-like strategy, reconfiguring the arm into a stiffened *quasi*-articulated structure. These movements were studied by analyzing the kinematics of the movements of specific points along the arm which display several stereotypical characteristics. Electromyographic recordings and detailed biomechanical simulations assisted in revealing common principles which reduce the complexity associated with the control of these movements. The travelling bend, used in arm extension movements, was found to be associated with a propagating wave of muscular activations, where simple adjustments of the excitation levels at the initial stages of the movement can set the velocity profile of the whole movement. Recently, a soft robotic arm inspired by the octopus arm has been designed in order to reproduce the octopus tentacle motor performance and to examine the possibility for the implementation of motor control principles identified in the octopus as part of its control (Laschi et al., 2009; Calisti et al., 2011).

However, describing the movements of specific points along the arm is insufficient for analyzing the full complexity of octopus arm movements. To determine whether the kinematics of octopus arm movements can be described by a reduced set of motion primitives requires analysis of different types of arm movements and of the shape of the entire arm as it moves through space. Motion primitives can be regarded as a minimal set of movements, which can be combined in many different ways giving rise to the richness of vertebrate and invertebrate movement repertoires and allowing motor learning of new skills (Flash and Hochner, 2005; Bizzi et al., 2008). Motor primitives have been inferred at various levels of the motor control system. Sub-movements were shown to be combined at the kinematic level (Krebs et al., 1999; Rohrer et al., 2002), a reduced set of static force field underlie controlling arm posture (Mussa-Ivaldi and Bizzi, 2000; d'Avella et al., 2003, 2006), while movement dynamics can be learned through a flexible combination of dynamic primitives (Thoroughman and Shadmehr, 2000). Dynamical movement primitives were also used to model attractor behaviors of autonomous non-linear dynamical systems and rhythmic movements (Ijspeert et al., 2002, 2013), and discrete and rhythmic movement elements were used to investigate single-joint and multi-joint motor behaviors (Sternad et al., 2000; Sternad and Dean, 2003). Inferring motion primitives from octopus arm movements may help understand underlying principles and kinematic optimal measures, and provide new understanding of how the nervous system in muscle hydrostats handles the complexities associated with the control of hyper-redundant arms. This may also facilitate designing control systems for hyper-redundant robotic manipulators.

Here we refer to the behavioral level and aim at describing octopus arm behaviors as being composed of elementary kinematic units to which we also refer to as motion primitives (Flash and Hochner, 2005). We believe that identifying basic kinematic patterns is the first step in further investigating the existence of primitives also at the control, movement dynamics, and muscle activation levels as well as the neural control levels as was demonstrated in earlier studies of the octopus motor system (Gutfreund et al., 1996, 1998; Sumbre et al., 2001, 2005, 2006; Yekutieli et al., 2005a,b). As was discussed in Flash and Hochner (2005) elementary building blocks may exist at all the above levels of motor representation but the most immediate and direct way is to search for elementary units at the kinematic level. Movement strokes with specific spatial and temporal features and submovements were shown to successfully describe both periodic and discrete motions and were indicated as plausible building blocks of human and monkey movements (Sosnik et al., 2004; Polyakov et al., 2009). Furthermore, in robotics research locomotion trajectories for a humanoid robot were constructed based on kinematic motion primitives derived from humans' locomotion trajectories (Moro et al., 2011, 2012). Relations between behavioral and control levels were suggested in different earlier studies, for example: hand trajectories of stroke patients were shown to be composed of submovements with velocity primitives obeying the minimum jerk model (Flash and Hogan, 1985) whose number was found to decrease as the patients gained better control of their limb (Rohrer et al., 2004), simple curved two-dimensional trajectories that follow the two-third power law (Lacquaniti et al., 1983) were described by means of parabolic units and corresponded to neural activation states identified using a hidden Markov Model (Polyakov et al., 2009). Another example consists of grasping and object manipulation movements described as arising from well-coordinated combinations of basic motor actions-arm transfer and hand shaping (Jeannerod, 1994).

An algorithm for 3D tracking and analysis of octopus arm movements (Yekutieli et al., 2007; Zelman et al., 2009) enabled us to create a large database of many types of modeled octopus arm movements. Here we describe a new framework allowing the extraction of kinematic units from these reconstructions. The octopus arm movements were represented by a pair of surfaces describing the curvature and torsion values of the arm. 2D Gaussians for each surface were extracted such that each Gaussian represented the characteristic shape of the curvature or torsion along a section of the octopus arm during some time interval. We found that Gaussian functions generally fit quite well the continuous form of the configurations that the octopus arm can take with respect to both the time and the arm index dimensions: the curvature and torsion values were observed to change smoothly along the arm length for any quasi-static arm configuration, and the magnitude of curvature or of torsion at any specific point was gradually changing with time during the movement. Gaussian-like functions were previously used in composing hand velocity (Thoroughman and Shadmehr, 2000) and limb position profile (Hwang et al., 2003).

The resulting Gaussians were divided into clusters whose centroids defined kinematic units, and each movement was represented as a weighted combination of such units. These kinematic units can be used to form a language of motion primitives, allowing characterization and representation of a large repertoire of octopus arm movements. We show how these kinematic units can be used to classify octopus arm movements into meaningful groups. Understanding how kinematic primitives can be utilized and combined can greatly contribute not only to studies of motor control in octopus arms and other hyper-redundant appendages but can also provide a deeper understanding of motor control systems in general.

## Methods

The analyzed octopus arm movements in our study were performed by four specimens of *Octopus vulgaris*, weighing 200 (female), 200 (male), 450 (male), and 470 (female) g. The animals were maintained in 50 × 40 × 40 cm tanks containing artificial seawater. The water was circulated continuously in a closed system through a biological filter of Orlon, gravel and coral dust. Water temperature was held at 16°C in a 12 h light/dark cycle. Prior to the video recording sessions, the animals were moved to a bigger glass tank (80 × 80 × 60 cm) with a water temperature of 18°C.

### Spatio-temporal representation of movement as a pair of curvature and torsion surfaces

Since the octopus arm displays no well-defined landmarks, a skeletal representation can be naturally used to model the octopus arm using curves which prescribe its virtual backbone. The backbone was found using a “grass fire” algorithm that extracts the middle line of the arm: first the contour of the arm is separated into two sides, dorsal and ventral, from base to tip. Then two distinct waves are initiated from the two sides of the contour and are propagated at an equal speed inward. The set of points where the wave fronts collide is the midline (Yekutieli et al., 2007).

**Figure 1:**
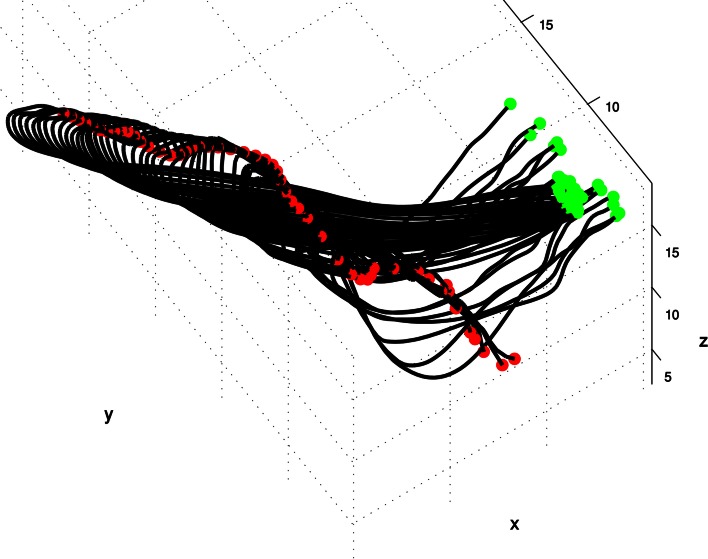
**A spatio-temporal profile of an extension movement shown as a sequence of the virtual backbone of quasi-static arm configurations.** The presented 3D curves are the result of the reconstruction process in which the virtual backbone prescribing the octopus arm is detected (see section spatio-temporal representation of movement as a pair of curvature and torsion surfaces). The virtual backbone was found by a “grass-fire” algorithm, green and red points mark the base of the arm (that was aligned between sequential images using textural cues) and the tip respectively (Yekutieli et al., 2007).

The reconstructions of octopus arm movements result in a sequence of 3D curves prescribing the virtual backbone of the octopus arm as its configuration changes during the movement. Figure 1 presents an extension movement as a sequence of 60 3D curves that prescribe the virtual backbone during the movement. For each curve, green, and red points mark the base of the arm (that was aligned between sequential images using textural cues) and the tip, respectively. Given a sequence of *m* 3D curves as an input, we wished to construct a pair of surfaces describing the values of the curvature and torsion along these curves. Since arm configurations were reconstructed from video records whose sample rate was 50 frames/s, the smoothness of the motion between consecutive configurations of a movement was guaranteed, and a spline function was used to smooth noisy points as necessary (Yekutieli et al., 2007; Zelman et al., 2009).

Each 3D curve was first represented by (*n* = 100) sample points uniformly distributed along the curve. This 3D curve was then approximated by a cubic smoothing spline constructed of piecewise third-order polynomials passing through the *n* sample points. Approximation was achieved, by considering both the smoothness of the spline and the distance between the spline and the sample points. Formally, given the data site *x*(*j*) and the corresponding data values *y*(*j*) for *j* = 1, …, n, the cubic smoothing spline *f* minimizes:

$$p\sum_{j\;=\;1}^{n}{\left|y(j)-f(x(j))\right|}^{2}+(1-p)\int {\left|D^{2}f(t)\right|}^{2}dt,$$

where the integral over the second derivative of *f* is over the smallest interval containing all the entries of *x*. The smoothing parameter *p* defines the tradeoff between the success in approximating the data points and the smoothness terms.

We then calculated the curvature (κ) and torsion (τ) values for the *n* sample points along each of the 3D curves. The curvature was calculated using the circle passing through three successive points as an approximation of the osculating circle to the curve at the middle point. This is formally described by Calabi et al. (1998): Let *A*,*B*,*C* be three successive points on the curve *C* such that the Euclidean distances are *a* = *d*(*A*,*B*), *b* = *d*(*B*,*C*), *c* = *d*(*A*,*C*). Let Δ denote the area of the triangle whose vertices are *A*,*B*,*C*, and let $s=\frac{1}{2}(a+b+c)$ denote its semi-perimeter, so that $\Delta =\sqrt{s(s-a)(s-b)(s-c)}$. Then the radius of the circle passing through the points *A,B,C* is computed leading to the formula for its curvature:

$$\text{κ}(A,B,C)=4\frac{\sqrt{s(s-a)(s-b)(s-c)}}{abc}.$$

In this study we will use the word curvature as the inverse of the radius of curvature.

The torsion along a 3D curve, defined as $\tau =\frac{d\theta }{dt}$, was calculated for a pair of successive points as the angle between the normals to the planes defined by the successive triangles corresponding to these points, divided by the distance between the points (Boutin, 2000). Let *A,B,C,D,E* be five successive points on the curve such that ${\hat{n}}_{ABC}$ and ${\hat{n}}_{CDE}$ are the normals to the planes defined by *A,B,C* and *C,D,E* respectively, and the Euclidean distance between points *B* and *D* is *d*(*B*,*D*). Then the torsion at point *C* is calculated as:

$$\text{τ}\left(C\right)=\frac{\cos^{-1}({\hat{n}}_{ABC}\cdot {\hat{n}}_{CDE})}{d(B,D)}.$$

Finally, the curvature and torsion values calculated for a sequence of 3D curves were represented on two surfaces that separately described the curvature and torsion as a function of time and arm index. The result was a pair of smooth and normalized curvature and torsion surfaces, such that a single arm movement was compactly represented by a pair of *n* by *n* matrices. This representation was invariant to rotation and translation in a Cartesian coordinate system, as curvature and torsion measures are intrinsic (i.e., they do not depend on the orientation and position of the arm in 3D space).

**Figure 2:**
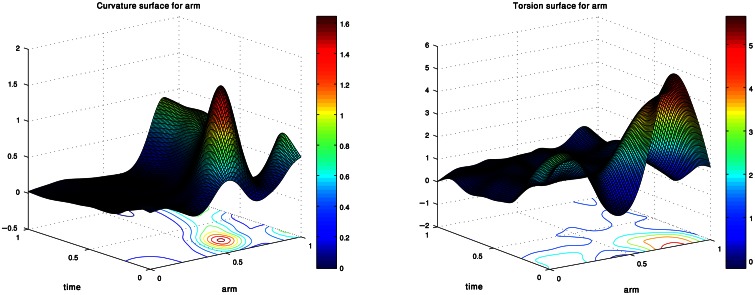
**Curvature and torsion surfaces extracted for the movement shown in Figure 1.** The values are given as a function of the arm index and time.

Figure 2 presents the curvature and torsion surfaces for the extension movement presented in Figure 1. The relatively high values of the curvature surface generally describe propagation of a bending section from the middle of the arm toward the tip. The decrease in the torsion values as the movement proceeded means that the arm configuration became relatively planar.

### Surface decomposition using GMM

Gaussian Mixture Model (GMM) is a statistical method for density estimation and data clustering (McLachlan and Peel, 2000). In this model a Gaussian fitting method can be used to approximate a function of one variable by a weighted sum of 1-dimensional Gaussians. As GMM is a generalized framework, it can approximate any multidimensional data by a set of multivariate Gaussians. The model uses an iterative process which optimizes the Gaussians' parameters by the Expectation Maximization (EM) algorithm (Xuan et al., 2001).

A function of one variable (*y* = *f*(*x*)) can usually be approximated by a mixture of 1D Gaussians, where each Gaussian is defined by its *mean* and *standard deviation*. In our case, we refer to a surface as a function of two variables *z* = *f*(*s,t*), where *z* stands for either the curvature or torsion values, and *s,t* refer to the arm index and time dimensions, respectively. We therefore use 2D GMM to approximate a surface by a weighted combination of 2D Gaussians. Specifically, a surface is approximated as:

$$z(s,t)=\sum_{i}w_{i}\cdot g_{\left[\mu_{i},\Sigma_{i}\right]}(s,t),$$

where *g* is a Gaussian defined by a 2 × 1 *mean* vector μ and 2 × 2 *covariance* matrix Σ, and *w* is the Gaussian weight. The Gaussian *g* is defined as:

$$g_{\left[\mu ,\Sigma \right]}(\vec{x})=\frac{1}{2\text{π}{\left|\Sigma \right|}^{1/2}}\exp\left(-\frac{{\left(\vec{x}-\mu \right)}^{T}\Sigma^{-1}(\vec{x}-\mu )}{2}\right)\text{​},$$

where $\vec{x}=|\frac{s}{t}|$. The *mean* vector μ corresponds to the position of the Gaussian center on the surface, and the two eigenvalues of the *covariance* matrix Σ correspond to the *standard deviation* of the 2D Gaussian. Its two eigenvectors correspond to the axes of the Gaussian with respect to a fixed coordinate system, such that the *covariance* matrix defines the shape and orientation of the Gaussian.

We also added a criterion to choose the right number of Gaussians into which each surface should be optimally decomposed, based on the Minimum Description Length (MDL) principle. The MDL descriptor to be minimized here is the Bayesian Information Criterion (BIC), as developed by Andrews and Lu (2001):

BIC=−2 ·L+d·log(n)

where *L* is the log likelihood of the mixture of Gaussians, *d* is the number of parameters in the model (number of degrees of freedom) and *n* is the number of observations in the sample. The BIC criterion allows choosing the most parsimonious model, i.e., the model which best describes the data with respect to the number of Gaussians it uses for the decomposition. [See also Bhat and Kumar (2010) for a more detailed derivation of the BIC formula].

**Figure 3:**
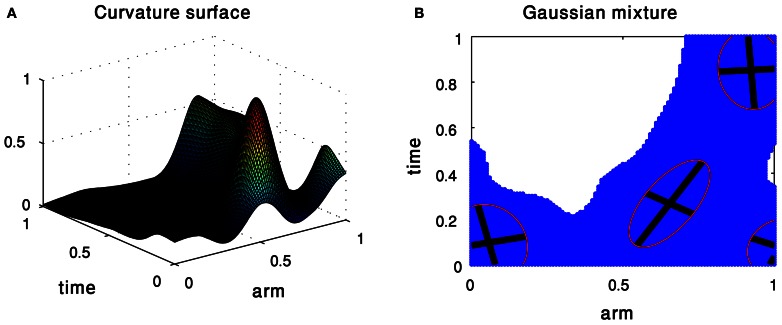
**Gaussian Mixture Model of the curvature surface of an extension movement. (A)** The input surface. **(B)** The resulting mixture of Gaussians.

Figure 3 shows the approximation of a curvature surface by a weighted combination of four Gaussians. Intuitively, each Gaussian can be illustrated as a hill, whose center, shape, orientation, and height are defined by the Gaussian parameters. The decomposition into 2D Gaussians allows us not only to explore Gaussians as possible units enabling to define the kinematics of octopus arm movements, but also to compactly represent a surface as a weighted sum of 2D Gaussians.

### Clustering algorithm

The GMM allows us to decompose octopus arm movements into curvature and torsion 2D Gaussians which describe their kinematics. We refer to each of the resulting Gaussians as a data point, whose dimension corresponds to the number of parameters defining a Gaussian (section surface decomposition using GMM). To cluster these points (i.e., the 2D Gaussians) into meaningful groups we used the *kmeans* clustering algorithm which is an unsupervised clustering method. The output of the *kmeans* algorithm is *k* disjoint clusters, where each cluster includes a different number of points and is represented by a centroid that can be regarded as the average of all the points assigned to the cluster. *kmeans* uses a two-phase iterative algorithm to yield a clustering result which minimizes the point-to-centroid distances summed over all *k* clusters.

The distance usually employed for *kmeans* is the Euclidean distance (Hastie et al., 2009), but here we want to improve the clustering from two points of view. First, we designed a Weighted Euclidean Distance, i.e., for each of the parameters of the Gaussians—center, shape, area, and orientation, we separately computed the Euclidean distance among the different elements of the sample. We then got four distances, each being related to one of the four parameters. The quantity to be minimized in the *kmeans* algorithm at each step is then an average of these four distances. Second, we used the Gap-Statistics (Tibshirani et al., 2001) as a criterion of the optimal number of clusters to be used. Gap-Statistics compares the within-clusters distance of the distribution (given by *kmeans*) to the within-distance W^*^_kb_ of a Monte–Carlo sample drawn within the range of the reference distribution. This criterion was used for example by Ben-Hur et al. (2002) and Pedersen and Kulkarni (2006). The idea of this approach is thus to compare the graph of log(*W*_*k*_) (log of the within-cluster distance) with its expectation under an appropriate null distribution. The mathematical rationale of this approach is explained in greater detail by Tibshirani et al. (2001). Defining *B* as the number of generated data sets, the Gap-Statistics is expressed as:

$$\text{Gap}\left(k\right)=1/B\sum_{b\;=\;1}^{B}\log\left(W_{kb}^{*}\right)-\log(W_{k})$$

The optimal number of clusters is the minimal *k* which gives a local maximum of the Gap.

## Results

Our data set consisted of 60 reconstructions of octopus arm movements that included *extension* movements. Extension in the octopus arm is generally characterized by a bend propagating along the arm (Figure 11). Some of these extension movements were preceded by initialization movements, referred to as *pre-extension* movements, in which the octopus arm moved from an initial random position to a configuration that seemed to be ideal for the extension (Figure 12).

Careful examination of the video sequences allowed us to define start- and end-points of the extension phase as characterized in earlier studies (Gutfreund et al., 1996, 1998). In an extension movement, a bend is created usually near the base of the arm and is propagated along the arm toward the tip where it vanishes, while the base of the arm points in the direction of propagation. A pre-extension movement is generally defined as a movement in which an arbitrary configuration of the arm is reconfigured to an initial extension configuration. Based on these observations we initially divided our data into 25 pre-extension and 60 extension movements. In order to characterize sets of kinematic units and determine synthetic rules allowing reconstruction of the observed movements, we next decomposed the movements into curvature and torsion Gaussian units (as defined in section surface decomposition using GMM) and analyzed these units as described below. Since each movement was defined by a specific combination of kinematic units, we could classify the movements into sub-groups, such that all the movements in a sub-group were defined by a combination of similar kinematic units. In order to explain the different phases of the movement analysis as clearly as possible we focus here mainly on the group of extension movements.

### Decomposition and clustering of kinematic units

Curvature and torsion surfaces were extracted for all the octopus arm movements in our database (see section spatio-temporal representation of movement as a pair of curvature and torsion surfaces). The curvature and torsion values at the tip of the arm could be very high (and sometimes noisy) relative to the values along the proximal and middle parts of the arm and were, therefore, analyzed separately. The resulting surfaces were approximated using the GMM, yielding decompositions of each curvature/torsion surface into 2D Gaussians units (see section surface decomposition using GMM). These units were found to naturally describe the surfaces, as each unit essentially described bending or torsion along a defined section of the arm and its movement along the arm as function of time.

A set of 2D Gaussians was assembled as a set of kinematic units separately for the pre-extension and extension movement groups. A set of Gaussians can be variously clustered by referring only to a subset of the parameters defining the 2D Gaussians, namely the center location, size, shape and orientation (see section clustering algorithm). These parameters were easily extracted from the *mean* and *covariance matrix* of a 2D Gaussian; the coordinates of the Gaussian center on the surface (i.e., the time point and the arm index at which the Gaussian reached its maximum) were directly defined by the Gaussian *mean*. The orientation of the Gaussian (the angle between the Gaussian axes and the axes of the fixed coordinate system) was defined by the eigenvectors of the Gaussian *covariance matrix*. The projection of the Gaussian on the plane was an ellipse whose size and eccentricity were defined by the eigenvalues of the *covariance matrix* and the ratio between them. Finally, the relative influence of the Gaussian in the decomposition in which it participated was defined by its weight. The clustering results presented here were obtained by referring to the Gaussian's center location (Gaussian *mean*), Gaussian's shape (ratio between the eigenvalues of the Gaussian's *covariance matrix*), and Gaussian's weight.

**Figure 4:**
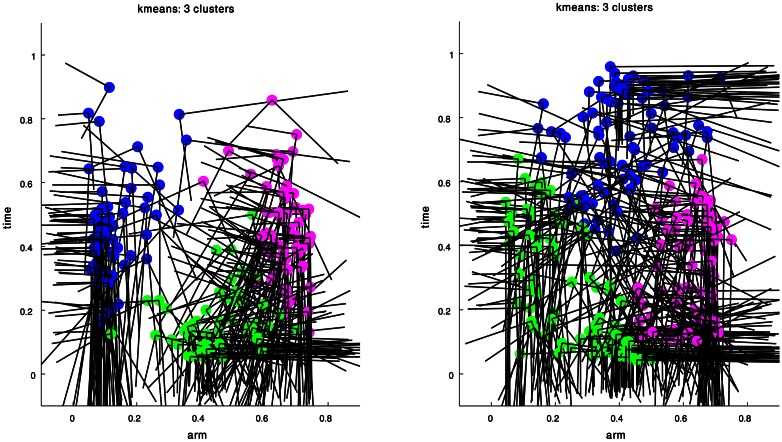
**Clustering results for the curvature (left) and torsion (right) Gaussians that were extracted from a group of 60 extension movements by GMM.** The arm index and time coordinates of each cluster centroid are given in Table 1.

Figure 4 presents the clustering results obtained for the curvature and torsion Gaussians of the 60 extension movements. Gaussians marked by the same color belong to a single cluster. Executing *kmeans* with the Gap-Statistics method (section clustering algorithm) resulted in three clusters both for the curvature and torsion Gaussians. Coordinates of the centroids of the various clusters are presented in Table 1.

**Table 1:** **The arm index and time coordinates of the cluster centroids shown in Figure 4**.

**Centroids of curvature Gaussians**			**Centroids of torsion Gaussians**		
**Cluster no.**	**Arm index coordinate**	**Time coordinate**	**Cluster no.**	**Arm index coordinate**	**Time coordinate**
1 (green)	0.4627	0.1574	1 (green)	0.2687	0.2463
2 (magenta)	0.6647	0.4502	2 (magenta)	0.6245	0.3288
3 (blue)	0.1355	0.4543	3 (blue)	0.4061	0.7207

These results essentially suggest that all the Gaussians composing the curvature and torsion surfaces of the extension movements can be classified into three types according to the values of the Gaussian's center location and shape. Explicitly, the blue curvature cluster (Figure 4 left) represents curvature Gaussians defining curvature along the proximal section of the arm during the movement. Examining the orientation of these Gaussians as defined by the eigenvectors of their *covariance* matrices shows that there was a relatively small angle between each of the Gaussian axes and the axes of the arm-index—time coordinate system (Table 2), that is, the internal Gaussian axes almost aligned with the direction of the arm-index and time axes. This characteristic means that the section of the arm to which these Gaussians relate did not change during the movement; we therefore term them “fixed” Gaussians. We suggest that these Gaussians correspond to movements used to aim the base of the arm toward a target point during the extension movement (see discussion below).

**Table 2:** **The mean (μ), median μ_1_/2) and standard deviation (σ) for the orientation values (in degree) of the resulting curvature and torsion clusters shown in Figure 4**.

**Orientation of curvature Gaussians**				**Orientation of torsion Gaussians**			
**Cluster no.**	**μ**	**μ_1_**/**2**	**σ**	**Cluster no.**	**μ**	**μ_1_**/**2**	**σ**
1 (green)	45.8	28.8	42.6	1 (green)	21.4	8.3	31.1
2 (magenta)	40.4	31	35.9	2 (magenta)	32.2	22.3	30.5
3 (blue)	19.9	11.2	24.2	3 (blue)	39.4	29.2	35.7

The green and magenta clusters represent curvature Gaussians that travel toward the tip of the arm during the extension and are probably associated with the main characteristics of the bend propagation in extension movements. The mean orientation of each of these clusters is significantly larger compare with the blue cluster that refers to the base of the arm (Table 2). A similar interpretation is valid for the resulting clusters of the torsion Gaussians, where the green and magenta clusters refer to torsion Gaussians in the early stage of a movement, and the blue cluster refers to torsion Gaussians at the end of movement. Table 2 presents the median, mean and standard deviation of the orientation values of the resulting clusters. These findings are supported by earlier analyses and simulations of the stereotypical characteristics of an extension movement (Gutfreund et al., 1998; Yekutieli et al., 2005b).

### Synthesizing arm behaviors from kinematic units

The 2D Gaussians were clustered by the *kmeans* algorithm based on their mean vector and covariance matrix values. The centroid point of each cluster represented the center of the cluster, i.e., the point giving the minimum sum of distances from it to all the data points in that cluster. Since the *mean* vector and *covariance* matrix which define a Gaussian as a data point also apply to the centroid point, a centroid point essentially defines a representative Gaussian for its cluster. Each of these Gaussians has a unique position, size and orientation, thus uniquely defining the octopus arm movement in 3D space.

**Figure 5:**
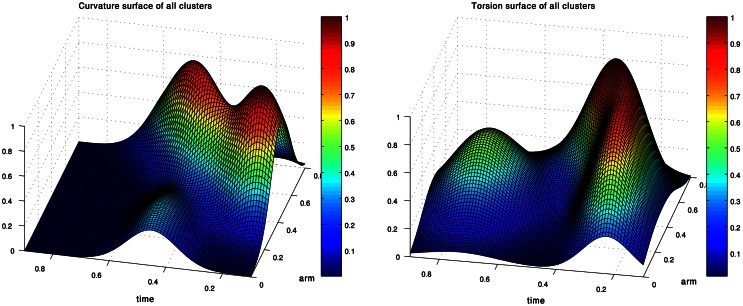
**The curvature (left) and torsion (right) centroid Gaussians of the extension group of movements.** Each Gaussian is essentially the centroid of one of the clusters in Figure 4.

**Figure 6:**
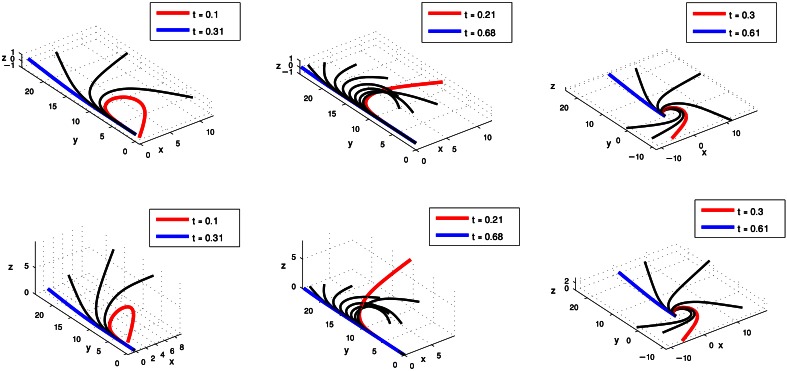
**Simulations of octopus arm behaviors in 3D space defined by the curvature and torsion kinematic units which were extracted for the extension movement group.** These behaviors show the characteristics of extension movements—directing the base toward a target, initialization, and propagation of the bend.

We therefore consider the curvature and torsion Gaussians defined by the resulting centroid points as kinematic units that can be used to generate a set of behaviors of the octopus arm. These are local time-dependent behaviors, since they refer to a specific section of the arm at a specific time during the movement. Figure 5 presents the three curvature Gaussians (left) and the three torsion Gaussians (right) defined by the centroid points of the clusters found for the extension movements (Figure 4). A curvature unit alone defines a planar arm behavior, as it defines a change in the curvature level along a section of the arm as a function of time, with a zero value for the torsion associated with the arm. Coupling a curvature and a torsion unit, such that both of them refer to a common section of the arm, defines a 3D behavior. However, a torsion unit on its own is meaningless since applying torsion on a straight line representing the backbone of the arm has no effect on its configuration. A torsion unit has no significant effect also when it is coupled with a curvature unit that refers to a different section of the arm. In general, *n*_*C*_ curvature units and *n*_*T*_ torsion units can define *n*_*C*_ · *n*_*T*_ 3D behaviors, and since the *n*_*C*_ curvature units define *n*_*C*_ planar behaviors where they are not coupled with any torsion unit, they can overall define *n*_*C*_ · (*n*_*T*_ + 1) behaviors. Figure 6 presents some of the behaviors that can be defined by the curvature and torsion kinematic units extracted for the extension group (Figure 5). They are shown as sequences of quasi-static configurations in 3D space, where the red, black and blue curves represent the initial, intermediate and final configurations in the sequence, respectively.

### Classifying octopus arm movements

The kinematic units (curvature/torsion Gaussians) extracted for the extension and pre-extension movement groups can be used further to classify movements in a given group into different sub-groups according to the mixture of Gaussians composing their curvature and torsion surfaces. Intuitively, movements that were decomposed into weighted combinations of similar kinematic units were classified into the same sub-group, as they were assumed to be characterized by a similar 3D behavior.

**Figure 7:**
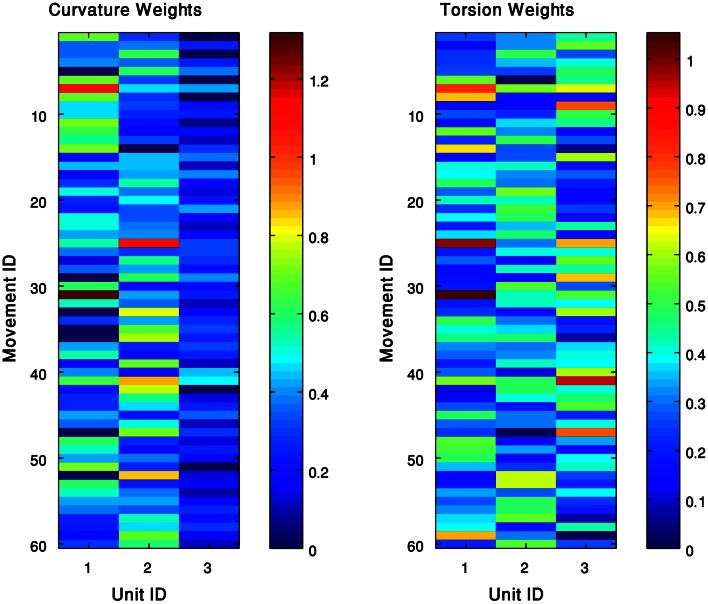
**The kinematic units can span the 60 extension movements.** Each movement is expressed as a weighed sum of curvature (**left**) and torsion (**right**) units. Clustering these weights results with patterns of weights, such that each one defines a movement prototype in this group.

We represented each of the movements in our data set by a weighted combination of the curvature and torsion kinematic units defined for the group of movements to which the movement belonged. That is, a movement *m* which was approximated by a pair of curvature (*C*) and torsion (*T*) surfaces *C* = ∑_*i*_ *w*^*C*^_*i*_ · *g*^*C*^_*i*_ and *T* = ∑_*j*_*w*^*T*^_*j*_ · *g*_^*T*^*j*_, was represented by a row vector of the weights: $\vec{w}=[\left\{w_{i}^{C}\right\}\text{​},\left\{w_{j}^{T}\right\}]$. We applied the *kmeans* algorithm (section clustering algorithm) to the set of vectors of weights corresponding to a group of movements, such that the input to the algorithm is a matrix of weights, where the rows correspond to the analyzed movements and the columns to the Gaussians that were previously identified as curvature and torsion units. Each row practically defines a movement as a weighted sum of the elementary Gaussian units (Figure 7). The *kmeans* algorithm separated the movements into clusters, such that movements belonging to the same cluster shared a similar pattern of weights. That is, a cluster consists of movements that can be spanned by a similar weighted sum of the available curvature and torsion units. We therefore refer to each of the resulting clusters as a sub-group of movements that share similar characteristics of their 3D behavior. The centroid point of each of the resulting clusters was considered a representative pattern of a weighted combination of kinematic units, which defined the behavior of the sub-group of movements in 3D space. We refer to these different behaviors as movement prototypes.

**Figure 8:**
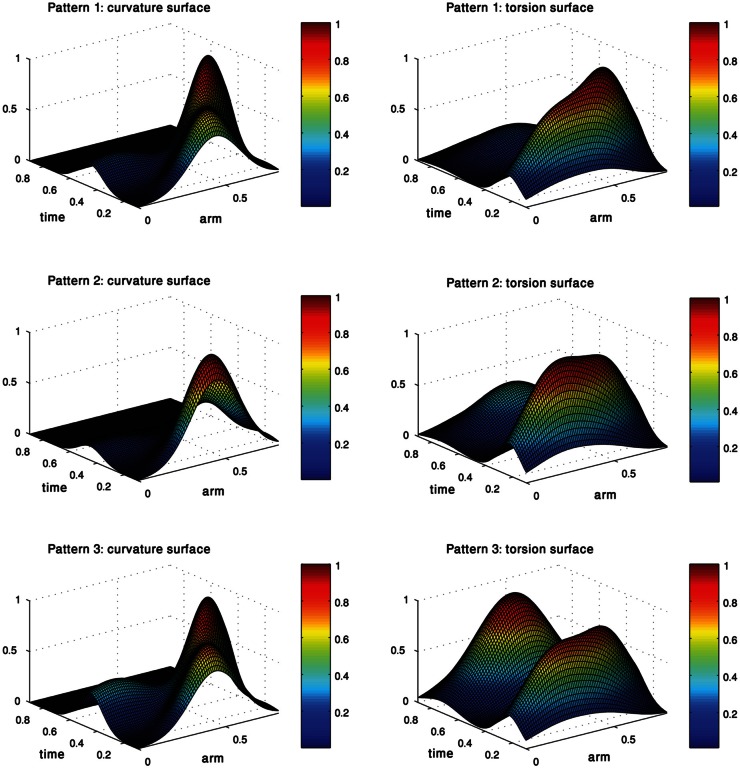
**Each pair of curvature and torsion surfaces defines one of the three prototypes of movements into which the extension movements were classified.** Each surface is essentially a weighted combination of the curvature/torsion Gaussians extracted earlier.

**Figure 9:**
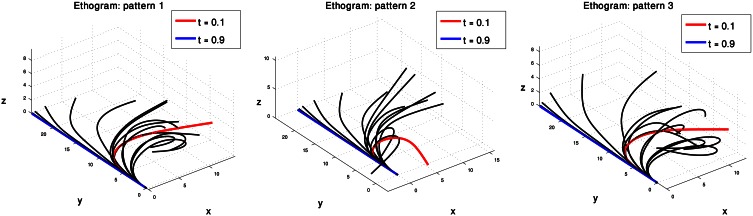
**Three prototypes represent the sub-groups into which the 60 extension movements were classified.** These prototypes, defined by three pairs of curvature and torsion surfaces (Figure 8) show the differences in the various extension movements. See text for further explanation.

Each of the three pairs of curvature and torsion surfaces presented in Figure 8 is defined by a weighted combination of kinematic units, corresponding to one of the extension sub-groups we found by the process just described. A pair of curvature and torsion surfaces defined a prototype which characterized one of the sub-groups by simulating a sequence of 3D curves whose curvature and torsion values corresponded to the values given by the curvature and torsion surfaces (Figure 9). Although the curvature surface of the first and third prototypes (Figure 8 top, bottom) share a similar topographic structure, the difference between their curvature surfaces defines prototypes with different characteristics. Relative to the first prototype, the third prototype (Figure 9 right) describes an extension movement in which a higher level of torsion is observed along with the propagating bend, causing the 3D configuration to deviate from the movement plane. Furthermore, the higher weight of the proximal curvature Gaussian results in a higher level of curvature along the base of the arm. The values presented by the torsion surface for the first prototype decrease during the second half of the movement, meaning that the configuration of the arm tends to become more planar as the movement progresses. For the first prototype (Figure 9 left) the arm section around the propagating bend, which takes higher curvature values, creates a loop during the initial phase of the extension movement. Compared to the first and third prototypes, in the second prototype (Figure 9 middle) the propagating bend starts with a higher curvature value and lower curvature values for the proximal section of the arm.

### Pre-extension results

**Figure 10:**
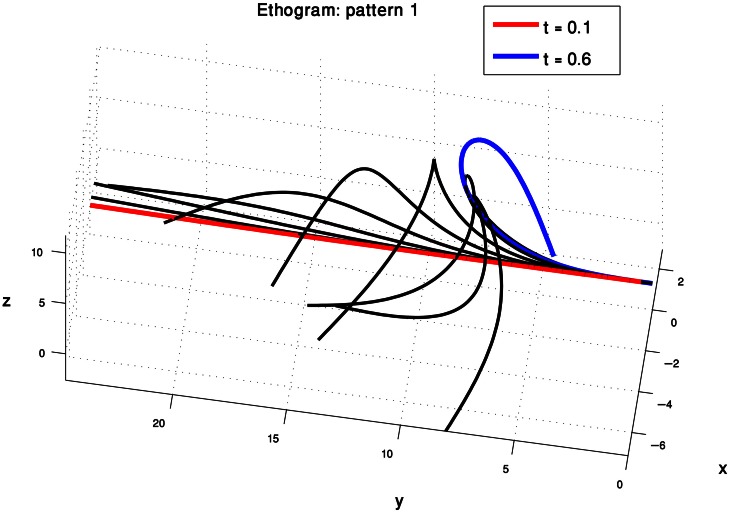
**One prototype was found to represent the pre-extension movements.** Pre-extension movements are intuitively understood as an initialization phase, during which the bend propagating during the extension phase is generated and the base of the arm is directed toward the target. The form of this prototype suggests that the movement is initialized by generating a new bend at the appropriate position by propelling the mid-section of the arm.

The analysis described above was also applied to the *pre-extension* movement group. The movements in this group refer to the actions that the octopus arm was observed to perform just before the extension phase has started. The well-defined time point in which the bend starts to propagate along the arm has been used to define the time at which a pre-extension movement ends. The kinematic units extracted for this group and the arm behaviors showed some similarities but also some unique characteristics. Figure 10 presents the single prototype that was found for pre-extension movements. It appears to represent the initializing phase of the arm, in which the base is directed toward a target and the bend (which is propagated during the extension) is generated. The initialization of the bend is achieved by generating movements corresponding to the curvature and torsion kinematic units on the same mid-arm section. Such dynamics may be associated with a minimal loss of energy due to interactions with drag forces. Computer simulations of the movements using the dynamic model of the octopus arm (Yekutieli et al., 2005a,b) will help to further explore and characterize this prototype with respect to muscle activation and energy expenditure.

## Discussion

By carefully watching the octopus arm movements in video sequences and identifying the time points bounding the extension phase, we were able to divide our data set of reconstructed arm movements into two main groups, *pre-extension* and *extension* movements. The analysis described here was applied separately to each of these groups but we have presented results mainly related to the extension group. Equivalent results for the pre-extension group are also available.

Instead of the common representation of octopus arm movements in 3D Euclidean space, we modeled each arm movement using pairs of curvature and torsion surfaces. These surfaces essentially describe the curvature and torsion values at the sampled points along the virtual backbone of the octopus arm as a function of time and arm index. Such pairs of curvature and torsion surfaces provide a compact description of arm configuration which is independent of the arm location in 3D space and is invariant to rotation and translation. Most importantly, this approach can be used to demonstrate the existence of kinematic units or motor primitives in octopus arm movements.

The characteristics of the surfaces led us to examine whether they can be meaningfully decomposed. We applied the GMM, suggesting the use of 2D Gaussians as building blocks approximating the curvature and torsion surfaces of a movement. These 2D Gaussians provided a mathematically quantified representation whose hilly shapes fitted well to the topographic characteristic of the surface. We thus have demonstrated a meaningful representation for octopus arm movements and a method GMM for decomposing the movements into well-defined building blocks (2D Gaussians) allowing us to further examine them as possible kinematic units. We have also applied an alternative method which decomposes a surface to its fundamental surfaces by analyzing the principal curvature values at each point on the surface. These parameters allow defining eight fundamental surfaces (e.g., peak, pit, ridge) that correspond to the topography of a surface (Yilmaz and Shah, 2005). Interestingly, we found the results extracted by this method to be very similar to those achieved using the GMM—the positions of the peak fundamental surfaces were highly correlated with the mean values of the positions derived using the 2D Gaussians.

Intuitively, being able to represent the arm by a 2D curvature Gaussian corresponds to the propagation of a bend point along a defined section of the arm and during a defined time interval. All the kinematic properties—the affected section of the arm, the time interval, the maximal curvature value and the velocity of propagation—are simply defined by the center location of the Gaussian, by its covariance matrix and by the weight assigned to this Gaussian. By clustering Gaussians with similar characteristics, we were able to characterize each cluster by its centroid and use the Gaussian representing the entire cluster as a stereotypical kinematic unit. We obtained a set of such curvature and torsion units for each of the pre-extension and extension movement groups. Curvature and torsion units were then combined to simulate new movements in 3D space and to examine whether the entire observed repertoire of complex 3D octopus arm behaviors can be spanned using the derived basic set of kinematic units. We found that patterns of weighted combinations of the kinematic units can be clustered into prototypes of movements in the pre-extension and extension phases, allowing classification of the movements into sub-groups.

The combinations of kinematic units which define the prototypes needs further investigation to reveal the principles underlying the execution of the different arm movements. Sumbre et al. (2001) have suggested that a relation between kinematic features and basic motor programs (embedded within the neural circuitry of the arm itself) greatly simplify the motor control of the octopus arm. In addition, a simple command producing a wave of muscle activation in a dynamic model was sufficient to replicate the kinematic characteristics of natural reaching movements (Yekutieli et al., 2005a). Specifically, it was found that natural extension movements can be generated by a dynamic model, in which a simple propagating neural activation signal is sent to contract muscles along the arm. In the model, the control of only two parameters fully specified the extension movement: the amplitude of the activation signal and the activation travelling time, such that different levels of activations can result in desired kinematics (Yekutieli et al., 2005b). We suggest that values of these two control parameters can be associated with the characteristics of the kinematic units extracted here. That is, the weight, shape, orientation, and size of a Gaussians can be related to the amplitude of the activation signal and the activation travelling time.

The relation between the kinematic units to the biomechanics of the octopus arm has to be examined (Feinstein et al., 2011). The arm morphology points to the dorsal group of the longitudinal muscles being much thicker than the ventral group, and both groups differ from the lateral groups. This anisotropy suggests that while bending movements to the left and right directions might be similar, this is not the case when comparing between upward, downward and sideward directions. The oblique muscles are composed of three pairs of helical bands, such that the handedness of the helix of one member of the pair is opposite to that of the other member of the same pair (Kier and Stella, 2007). This isotropy with respect to the arm axis supports that torsion toward the two different directions is applied in a similar manner.

**Figure 11:**
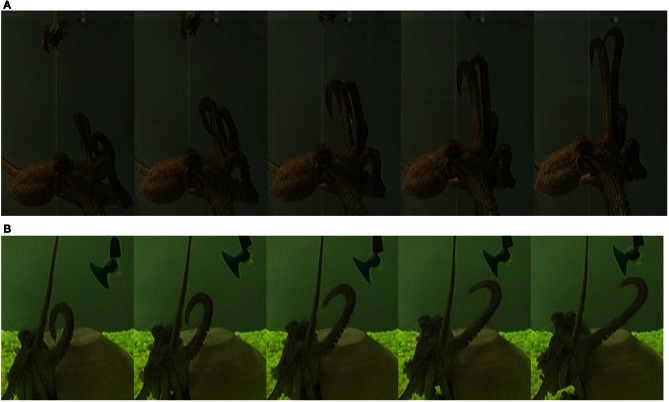
**Two extension movements.** The upper movement **(A)** was classified as prototype no. 2 of the extension group (Figure 9 middle), as a movement in which a highly curved bend was rapidly propagated toward a target, while the base of the arm stayed oriented with a fixed direction. The lower movement **(B)** was classified as prototype no. 1 of the extension group (Figure 9 left), as a movement in which the bend showed lower curvature values and moved relatively slowly toward the target while the direction of the base of the arm was not preserved. The movements progress from left to right in each panel.

The results from our analysis agree with our data on octopus arm movements. The extension movement shown in Figure 11A matched with prototype no. 2 of the extension group (Figure 9 middle), as a movement in which a highly curved bend along a relative short section of the arm propagated rapidly toward a target. The lower movement Figure 11B matched with prototype no. 1 of the extension group (Figure 9 left), as a movement in which the arm moved relatively slowly toward the target with relatively low curvature values for the propagating bend. The Gaussians referring to the main characteristic of extension movements strengthen the previous findings of Gutfreund et al. (1996) of a stereotypic profile of the position and velocity of the bend point. They suggested that the position of the bend in space and time is a controlled variable, which simplifies motor control. The travelling bend, associated with a propagating wave of muscle activation (Gutfreund et al., 1998), was simulated as a biomechanical mechanism in a dynamic model of the octopus arm (Yekutieli et al., 2005a).

**Figure 12:**
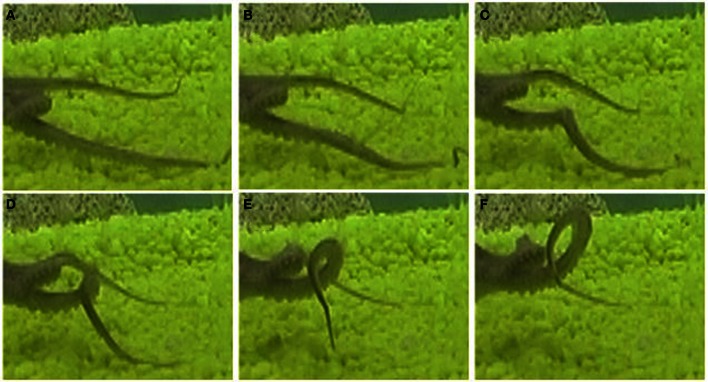
**A pre-extension movement as a sequence progressing from the upper left (A) to the lower right (F) frames.** A substantial manipulation, creating a bend and directing it toward a target, was applied to the initial configuration (upper left). This movement is matched with the prototype of the pre-extension group (Figure 10). Frame **(F)** presents a temporal configuration that matches the beginning of an extension movement.

Two pre-extension movements shown in Figure 12 matched with the prototype of the pre-extension group (Figure 10). A substantial manipulation of the initial arm configuration was involved by creating a bend in the arm and directing it toward the target. In this movement, curvature and torsion kinematic units are both applied on the mid-section of the arm during the pre-extension phase. These results demonstrate that the Gaussian description of movement primitives allows us to describe a complex motor behavior. Clearly, additional types of octopus arm movements other than the pre-extension and extension movements analyzed here are part of the motor repertoire of octopus behavior. While reconstruction and analysis of these other movements will probably reveal additional kinematic building blocks, we can expect that the general characteristics of octopus arm movements, mainly the smoothness in which curvature and torsion values vary along both the arm and the time dimensions will hold for other types of movements. Therefore, we believe that Gaussian functions could be efficiently used also in the decomposition and description of those movements.

Our results fit with Yekutieli et al.'s (2007) observations on the kinematic characteristics of the initiation of a reaching movement. The kinematic description is sufficiently rich for describing complex arm movements, although factors such as the biomechanics of the octopus arm (e.g., the different type of muscles and the constant volume constraint), water drag forces and energy expenditure also strongly influence the arm movement characteristics. For example, the perpendicular drag coefficient for an octopus arm is nearly 50 times larger than the tangential drag force coefficient. This most likely affects the preferred arm configuration during extension movements; only a small part of the arm is oriented perpendicularly to the direction of movement, minimizing drag (Yekutieli et al., 2005a). Yekutieli et al. (2005b) also found that the control of extension movements can be specified by the amplitude of the muscle activation signals and the activation travelling time. The primitives we suggest here can be used to further investigate the relation between the kinematic and muscle activation levels.

**Figure 13:**
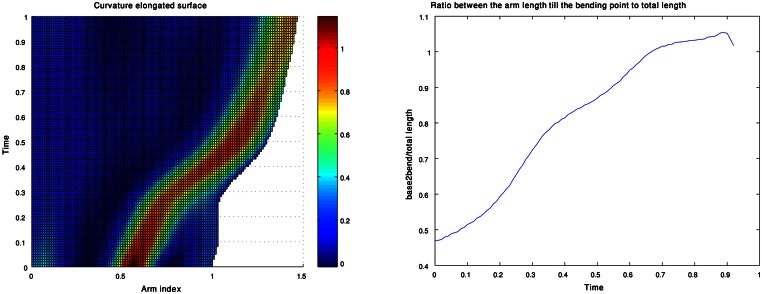
**A curvature surface which also refers to the elongation during an extension movement (left).** The values of the ratio between the lengths of the proximal section of the arm (from base to bend point) and the length of the entire arm as a function of time **(right)**.

In our analysis the curvature and torsion surfaces were extracted for arm configurations whose length has been normalized. Replotting the curvature and torsion surfaces while showing the actual arm length values as analyzed from live data (Figure 13 left) shows that the proximal section of the arm elongates during an extension (i.e., the section between the base and the bend point). This is demonstrated clearly in Figure 13 (right) which shows the ratio between the length of the proximal section to the length of the entire arm during an extension movement. Arm elongation has recently been shown to play a key role in the biomechanics and control of octopus arm movements (Hanassy, 2008). Modeling the travelling bend along extension movements based on the propagation of muscle activation and stiffening wave (Gutfreund et al., 1998), where co-contraction of both the longitudinal and transverse muscles pushes the bends forward (Yekutieli et al., 2005b). Therefore, different ratios between the activation levels of longitudinal to transverse muscle can be used to control the elongation of the arm along the proximal section between the base and bending point. Gaussian units, which were found in this study to describe the travelling bend during extension movements, will be further examined in order to support recent findings related to the biomechanics and control of the octopus arm.

Our analysis presents a possible language of kinematic primitives—2D Gaussians of either curvature or torsion which define and classify octopus arm behaviors by their different combinations. Constructing a taxonomy of possible movements for a species is one approach to the study of its behavior. To construct a taxonomy of octopus arm movements and to reveal how combinations of components result in a variety of behaviors Mather (1998) used components which consist of movements of the arm itself, the ventral suckers and their stalks, as well as the relative position of the arms and the skin web between them. Comparing similar movement taxonomies and ethograms (catalog of body patterns and associated behaviors) in the squid and various octopus species (Hanlon et al., 1999; Huffard, 2007; Mather et al., 2010) suggests that behaviors may be conserved throughout the evolution of these species. Our results identify a number of kinematic units, possible time-dependent units and sub-groups (Table 3). As more reconstructed octopus arm movements become available (Yekutieli et al., 2007; Zelman et al., 2009), we will better be able to use our analytical tools to define a comprehensive language of motor primitives that incorporates the underlying kinematic principles, thus enriching the ethogram and taxonomy of octopus arm behavior.

**Table 3:** **The analysis applied to each movement group yielded a number of kinematic units defining possible local-temporal 3D behaviors of the arm**.

**Group**	**Number of kinematic units**		**Number of possible behaviors**	**Number of sub-group prototypes**
	**Curvature**	**Torsion**		
Pre-extension	1	3	3	1
Extension	3	3	6	3

Each group was divided into a number of sub-groups which were represented by different movement prototypes.

Our analysis provides a new framework for research on the kinematics and control of any natural or mechanical flexible manipulator. Possible arm behaviors can be simulated by synthesizing new combinations of the extracted Gaussians. New primitives can be hypothesized and tested on dynamic models of the octopus arm and the resulting movements can be compared with live movements. This, in turn, may allow future studies of activation commands at the neural control level, which may then enable operation of a real flexible manipulator to perform specified goal-oriented tasks (Laschi et al., 2009, 2012; Calisti et al., 2011).

### Conflict of interest statement

The authors declare that the research was conducted in the absence of any commercial or financial relationships that could be construed as a potential conflict of interest.
